# Mental health and quality of life of patients with osteoarthritis pain: The sixth Korea National Health and Nutrition Examination Survey (2013–2015)

**DOI:** 10.1371/journal.pone.0242077

**Published:** 2020-11-12

**Authors:** Yura Lee, Sook-Hyun Lee, Sung Min Lim, Seung Ho Baek, In-Hyuk Ha

**Affiliations:** 1 Jaseng Hospital of Korean Medicine, Seoul, Republic of Korea; 2 Jaseng Spine and Joint Research Institute, Jaseng Medical Foundation, Seoul, Republic of Korea; 3 Department of Clinical Research on Rehabilitation, Korea National Rehabilitation Research Institute, Seoul, Republic of Korea; 4 Department of Korean Medicine, Dongguk University, Seoul, Republic of Korea; University of Mississippi Medical Center, UNITED STATES

## Abstract

**Objectives:**

This study aims to investigate the association between mental health and quality of life of osteoarthritis (OA) patients according to the site of pain.

**Design:**

Retrospective cross-sectional study

**Participants:**

Data of 22,948 participants of the sixth Korea National Health and Nutrition Examination Survey conducted from 2013–2015 were used.

**Outcome measures:**

Participants were asked if they had OA pain in the hip joint, knee joint, and lower back (yes/no) and whether they experienced anxiety or depression. The EQ-5D questionnaire was used to determine the quality of life of patients with hip, knee, and lower back OA. Multiple logistic regression analysis was performed after adjusting.

**Results:**

A total of 5,401 patients reported pain in the hip joint, knee joint, or lower back. The analysis showed significant relations between pain sites, mental health, and quality of life. First, more female patients with OA experienced stress and depression than males. Second, for males with OA, stress was reported in the order of: lower back > hip > knee, while pain and depression was reported in the order of: lower back > knee > hip (*p* < 0.05). For females with OA, stress was reported in the order of: knee > lower back > hip, while depression was reported in the order of: knee > lower back > hip. Third, considering quality of life, for males, hip joint pain had the greatest impact on quality of life and for females, knee joint pain had the largest impact (*p* < 0.001).

**Conclusions:**

For patients with OA, the effect on the mental health and quality of life differed according to sex and sites of pain. Therefore, this study confirms that pain sites, sex, mental health, and quality of life are independent risk factors when determining OA pain.

## Introduction

Osteoarthritis (OA) is a representative degenerative disease that commonly occurs in synovial joints. It has the highest incidence of all inflammatory diseases of the joints. Due to the gradual degeneration and damage to the articular cartilage, secondary damage occurs to other structures, such as the bones and adjacent ligaments, often leading to joint pain, deformity, and functional disability. In terms of radiographic features, pathological changes such as wear and tear of articular cartilage, osteophytes, and joint space narrowing can be observed, while clinical manifestations include joint pain, stiffness, decreased range of motion, crepitus, and mental disorders [1]. According to the 2010 Korea National Health and Nutrition Examination Survey (KNHANES), OA showed a prevalence of 5.7% among people in their 50s, three times that for people in their 60s, and five times that for those in their 70s and older. The prevalence of OA increases with age, and its incidence is higher in females than in males [2]. The prevalence of OA is expected to increase even more in the future, considering general population aging.

A representative symptom of OA is localized pain in the region where the OA occurs; it does not involve systemic symptoms. Other symptoms of OA include decreased range of joint motion accompanied by tenderness, swelling, and crepitus; these symptoms gradually progress [3]. Further, OA of the knee joint, for example, may be accompanied by gait abnormalities, while OA of the hip joint will affect posture and the formation of osteophytes will occur in the case of hand OA. Most people with OA experience mental health issues such as depression and anxiety and diminished quality of life, as well as physical problems such as difficulties with activities of daily living, falls, and possible substance abuse due to persistent pain and stiffness [4–6]. According to recent studies, the health-related quality of life of older adults with OA is significantly lower than that of older adults without OA [5–9]; furthermore, older adults with OA perceived their health status to be worse than those without OA [10]. OA also affects mental health, as prior studies report that older adults with OA are more depressed than those without OA [7,9,11]. Such mental health problems have the effect of worsening pain in older adults [12] and may increase the risk of suicide [13]. In their work, Bruce stated that OA and depression are closely related and that the physical symptoms of OA (fatigue, pain, and insomnia) and cytokines—inflammatory response substances—contribute to the development of depression [14]. The prevalence of OA is expected to continue to rise as modern society enters the era of aging populations. In these circumstances, multifaceted research on OA is essential.

As mentioned above, there are several studies examining the relationship between OA, quality of life, and mental health problems such as depression; however, no study has investigated the association between quality of life and mental health according to the sites affected by OA. In addition, the prevalence of OA varies significantly not only with age, but also with sex [15]. OA is five times more prevalent in females than in males [16]; furthermore, in males, OA occurs most frequently in the hip, whereas in females, the hand or knee joint show the most frequent development of OA. Therefore, this study selects three locations—the hip joint, knee joint, and lower back—of frequent OA occurrence in older adults [17] to compare and to analyze the association of quality of life and mental health according to the pain sites and according to sex.

## Materials and methods

### Study design and population

This study was conducted based on data from the sixth version of Korea National Health and Nutrition Examination Survey (KNHANES-VI) that was carried out in 2013–2015, an official survey conducted by a national agency in South Korea. KNHANES started in 1998 and was initially conducted every three years; however, since 2007, the survey has been taking place annually. The survey comprises a nationwide review of public health and nutrition and offers recognized representativeness and reliability. The purpose of the national survey is to understand the general characteristics, health behaviors, prevalence of chronic diseases, and nutritional intake status of the population and households in South Korea, and use the findings as a basis for public health policy. The current study was conducted with the approved use of source data from KNHANES-VI, which was obtained by submitting a request form for raw data and a summary of plans for use of the data via the KNHANES website, in accordance with the procedure for using raw data suggested by Korea Centers for Disease Control and Prevention (KCDC). Our report is a retrospective cross-sectional study.

**Fig 1:**
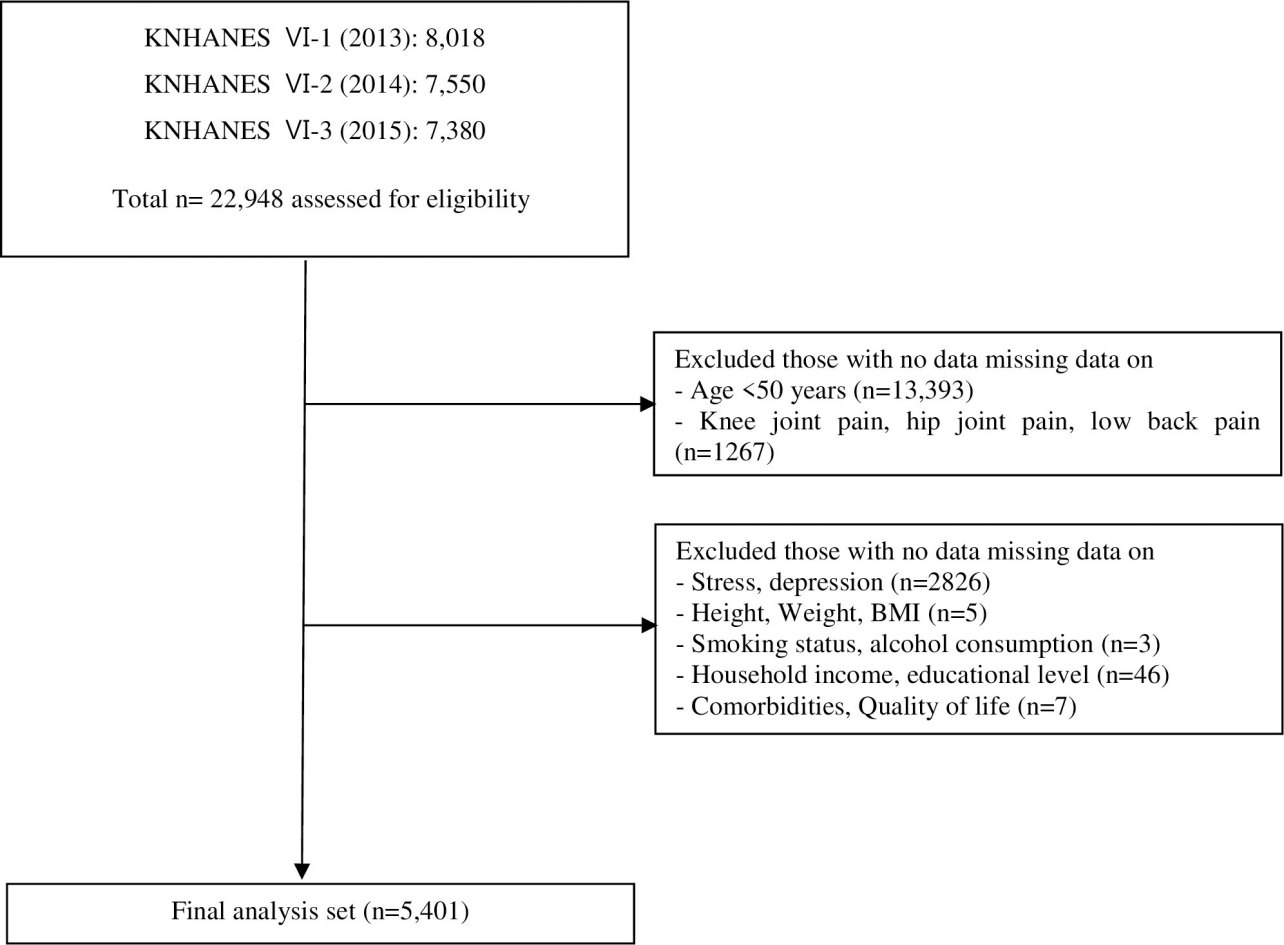
Subjects excluded from the analysis. KNHANES = Korean National Health and Nutrition Examination Survey; Comorbidities = angina, diabetes mellitus, hypertension, myocardial infarction, rheumatoid arthritis, and stroke.

For this study, 22,948 subjects who complained of hip pain, knee joint pain, and lower back pain were chosen from the KNHANES-VI; of these, 5,401 were included in the final analysis. Of the original 22,948 subjects, 13,393 subjects were younger than 50, 1,267 subjects had missing data on hip pain, knee joint pain, or lower back pain, and 2,826 subjects had missing data on stress and depression; these subjects were excluded. Furthermore, subjects who had missing data on height, weight, body mass index (BMI; five persons), smoking and alcohol consumption status (three persons), income and educational level (46 persons), or quality of life (seven persons) as well as those with comorbidities or chronic diseases were excluded. Consequently, this study was conducted for a total of 5,401 persons (Fig 1).

### Outcomes and other variables

Of the survey items of the KNHANES-VI, we used the following for analysis: age, sex, height, weight, BMI, educational level, household income level, number of comorbidities, smoking status, alcohol consumption, joint pain, depression and stress perception, and EQ-5D score.

Age, sex, educational level, and household income level were used for socio-demographic characteristics. Age was grouped into 50–59, 60–69, and 70 and older, while educational level was classified as “elementary school graduate or lower,” “junior high school graduate,” “high school graduate,” and “college graduate or higher.” Considering household income level, income was classified according to the quartiles of household income adjusted with equivalence scale, as presented in the KNHANES-VI data. These are “low,” “middle low,” “middle high,” and “high.”

For health-related characteristics, height, weight, BMI, number of comorbidities, smoking status, alcohol consumption, joint pain, depression and stress perception, and EQ-5D were used. For BMI, the values from anthropometric results were used as they were. Based on the World Health Organization’s standard, the formula of body weight (kg) / height^2^ (m^2^) was used for classification into underweight (BMI < 18.5), normal (18.5 ≤ BMI < 25), or obese (BMI≥25). Smoking status was classified into current smoker, ex-smoker, and non-smoker. Alcohol consumption status was classified into “none,” “one or fewer,” “two to three,” “four or more” alcoholic drinks consumed per week. The number of comorbidities comprise the number of diseases that have been diagnosed by a physician and that the patient is currently suffering from, excluding OA. In this study, the comorbidities were classified into types of diseases and the measure was used to identify whether the subject has been diagnosed with “angina,” “diabetes mellitus,” “hypertension,” “myocardial infarction,” “rheumatoid arthritis,” or “stroke” by a physician.

#### Measurement of the OA pain by site

The questions related to the sites of OA pain used in KNHANES-VI were analyzed using two items: whether they experienced pain for 30 days or longer in either of the pain sites (hip joint, knee joint, lower back) during the last three months and a respective joint pain scale. For the respective joint pain scale, subjects were asked to rate the average level of pain for each joint from 0–10, regardless of medication taken. We need to note that lower back pain was only assessed on whether there was lower back pain lasting more than 30 days during the last three months; there was no separate assessment of lower back pain level.

#### Assessment of mental health

Regarding the mental health-related questions used in KNHANES-VI, two items were used for assessment: “stress perception rate” and “rate of depression experience.” “Stress perception rate” indicates how many people responded that they perceived “very high” or “high” stress in their everyday lives, while “rate of depression experience” refers to the number of people who responded that they felt sadness or despair to the level of these feelings interfering with everyday living for two consecutive weeks or longer during the last year. In this study, the association (or relationship) between mental health and the different OA pain sites were assessed.

#### Assessment of quality of life

In the KNHANES-VI, the survey related to health-related quality of life was based on the EuroQol-5 Dimensions scale (EQ-5D). The EQ-5D is a well-known tool for the assessment of health-related quality of life by evaluating five dimensions: Mobility, Self-care, Usual activities, pain/discomfort, anxiety/depression. It was developed to assess individuals’ overall health status and is mainly used to evaluate the health-related quality of life of patients with chronic diseases. In this study, the values calculated by applying the KNHANES-VI KCDC weight model were used without modification. According to a study by Lim et al., the validity of the EQ-5D in relation to the assessment of the health-related quality of life of people with OA has been verified [18]. In this study, EQ-5D was used to assess subjects’ quality of life in relation to the OA pain sites (hip joint, knee joint, lower back).

### Statistical analysis

As the KNHANES-VI data are based on a complex sampling design, all the analyses in this study were complex sample analyses that consider weights, strata variables, and cluster variables. The differences in socio-demographic characteristics (sex, age, educational level, and household income level), lifestyle factors (alcohol consumption and smoking status), and disease and health factors (height, weight, BMI, comorbidities, depression and stress perception, and EQ-5D) according to the sites of pain were analyzed with a cross-tabulation analysis (χ2-test). Categorical variables were represented with ratios and percentages (*N*, %) and analyzed by chi-squared test. The continuous variables were presented in means and standard deviations and compared using a *t*-test. A multiple logistic regression analysis was used to calculate the odds ratios (OR) and 95% confidence intervals (CI) and to evaluate the associations between the OA pain sites, mental health, and quality of life. In addition, the association between OA pain sites and mental health and quality of life was sub-analyzed by sex (male/female). The statistical analysis was conducted using SPSS V25.0 (SPSS Inc., Chicago, IL, USA), and the significance level was set at *p* > 0.05.

### Ethics statement

KNHANES-VI was conducted by the KCDC and written consent was obtained from all participants. All survey protocols were approved by the institutional review board of the KCDC (approval numbers: 2013-12EXP-03-5C and 2015-01-02-6C). The data are available on the KNHANES website (http://knhanes.cdc.go.kr) and one can specify which annual reports are needed. This study was conducted with the approval of the IRB of JASENG, approval number: JASENG 2019-04-006.

## Results

### General characteristics

Table 1 presents the general characteristics of the study subjects. A total of 5,401 subjects reported pain in the hip joint, knee joint, and lower back. Among them, 556 subjects had hip joint pain and 4,845 subjects had no hip joint pain; 1,157 subjects reported knee joint pain while 4,244 subjects had no knee joint pain; and 1,246 subjects had lower back pain while 4,155 study subjects had no lower back pain. In all three groups, the number of subjects in the non-pain groups was higher than those in the pain groups.

**Table 1:** Characteristics of the study subjects according to the osteoarthritis pain site.

	Hip joint pain			Knee joint pain			Low back pain		
	Hip joint pain (n = 556)	Non-hip joint pain (n = 4,845)	*P*-value	Knee joint pain (n = 1,157)	Non-knee joint pain (n = 4,244)	*P*-value	Low back pain (n = 1,246)	Non-low back pain (n = 4,155)	*P*-value
**Age (years)** Mean±SD	66.0±0.46	61.8±0.17	p<0.001	65.1±0.36	61.5±0.18	p<0.001	65.7±0.32	61.2±0.18	p<0.001
50–59	137(30.6)	1907(49.3)	p<0.001	315(35.4)	1729(50.6)	p<0.001	332(33.5)	1712(51.3)	p<0.001
60–69	183(30.4)	1578(27.9)		359(28.1)	1402(28.2)		363(26.2)	1398(28.7)	
≥39	236(39.0)	1360(22.8)		483(36.5)	1113(21.2)		551(40.3)	1045(20)	
**Sex**, n(%)									
Male	122(22.7)	2168(48.8)	p<0.001	289(27.7)	2019(51.1)	p<0.001	328(28)	1980(51.3)	p<0.001
Female	434(77.3)	2659(51.2)		868(72.3)	2225(48.9)		918(72)	2175(48.7)	
**BMI (kg/m**^**2**^**)** Mean±SD	24.6±0.15	24.2±0.05	p<0.001	24.8±0.11	24.0±0.54	p<0.001	24.5±0.11	24.1±0.05	p<0.001
Low	15(2.4)	121(2.2)	0.129	19(1.3)	117(2.5)	p<0.001	32(2.6)	104(2.2)	0.031*
Normal	312(56)	2956(60.8)		633(55.3)	2635(61.7)		701(56.4)	2567(61.5)	
Obese	229(41.6)	1768(37)		505(43.4)	1492(35.8)		513(41.0)	1484(36.4)	
**Smoking status**, n(%)									
Non-smoker	412(72.4)	2862(55.8)	p<0.001	847(71.3)	2427(53.8)	p<0.001	886(68.7)	2388(54.2)	p<0.001
Ex-smoker	89(17.5)	1276(27.3)		186(16.8)	1179(28.8)		231(19.8)	1134(28.2)	
Current smoker	55(10.1)	707(16.9)		124(11.9)	638(17.4)		129(11.5)	633(17.6)	
**Alcohol consumption (day/week),** n(%)									
None	290(52.4)	1853(35.3)	p<0.001	575(48.4)	1568(33.9)	p<0.001	631(49.7)	1512(33.4)	p<0.001
≤1	144(25.2)	1199(24.3)		284(23.8)	1059(24.6)		301(23.1)	1042(24.8)	
2–3	97(17.6)	1396(31.3)		229(21.4)	1264(32.2)		246(21.5)	1247(32.3)	
≥4	25(4.8)	397(9.1)		69(6.4)	353(9.3)		68(5.7)	354(9.5)	
**Household income,** n(%)									
Low	251(43.0)	1365(25.6)	p<0.001	510(41.6)	1106(23.6)	p<0.001	545(42.1)	1071(23.2)	p<0.001
Low–moderate	136(24.1)	1279(25.2)		297(24.4)	1118(25.2)		310(23.8)	1105(25.4)	
Moderate–high	91(17.4)	1072(23.4)		198(18.1)	965(24.1)		209(17.5)	954(24.4)	
High	78(15.5)	1129(25.8)		152(15.9)	1055(27.1)		182(16.6)	1025(27.0)	
**Educational level,** n(%)									
Elementary school or lower	38(8.0)	734(16.5)	p<0.001	741(59.6)	1563(33.5)	p<0.001	764(57.7)	1540(33.6)	p<0.001
Middle school	79(15.2)	1322(29.4)		189(18.2)	735(17.3)		187(15.5)	737(18.0)	
High school	86(16.0)	838(17.6)		176(17.1)	1225(30.9)		204(18.4)	1197(30.8)	
College or higher	353(60.8)	1951(36.5)		51(5.1)	721(18.3)		91(8.5)	681(17.6)	
**Comorbidities,** n(%)									
**Angina**									
No	521(94.0)	4674(97.0)	0.003**	1094(95.5)	4101(97.1)	0.015*	1172(94.6)	4023(97.3)	p<0.001
Yes	35(6.0)	171(3.0)		63(4.5)	143(2.9)		74(5.4)	132(2.7)	
**Diabetes mellitus**									
No	452(81.1)	4114(85.8)	0.013*	930(79.6)	363(86.8)	p<0.001	999(79.5)	3567(87.0)	p<0.001
Yes	104(18.9)	731(14.2)		227(20.4)	608(13.2)		247(20.5)	588(13.0)	
**Hypertension**									
No	281(52.7)	2950(64.1)	p<0.001	595(53.9)	2636(65.4)	p<0.001	675(55.8)	2556(65.1)	p<0.001
Yes	275(47.3)	1895(35.9)		562(46.1)	1608(34.6)		571(44.2)	1599(34.9)	
**Myocardial infarction**									
No	546(98.7)	4763(98.3)	0.451	1132(98.1)	4177(98.4)	0.481	1219(98.1)	4090(98.4)	0.503
Yes	10(1.3)	82(1.7)		25(1.9)	67(1.6)		27(1.9)	65(1.6)	
**Rheumatoid arthritis**									
No	515(92.1)	4725(97.7)	p<0.001	1083(93.5)	4157(98.1)	p<0.001	1190(95.2)	4050(97.7)	p<0.001
Yes	41(7.9)	120(2.3)		74(6.5)	87(1.9)		56(4.8)	105(2.3)	
**Stroke**									
No	519(94.5)	4629(95.8)	0.174	1090(94.9)	4058(95.9)	0.225	1165(94.2)	3983(96.1)	0.016*
Yes	37(5.5)	216(4.2)		67(5.1)	186(4.1)		81(5.8)	172(3.9)	

Values are presented as mean ± SD or number (%). BMI (body mass index) was categorized into low (<18.5 kg/m^2^), normal (18.5–24.9 kg/m^2^), and obese (25.0 kg/m^2^). Household income level was calculated by dividing the total household monthly income with the obtained levels then grouped into quartiles. Educational level was divided into the following four groups: Elementary school or lower (≤6 years), middle school (7–9 years), high school (10–12 years), and college or high (≥13 years). P-values were calculated using Chi-square test or T-test.

*p<0.05

**p<0.001.

First, in terms of socio-demographic factors, the mean age of the OA pain group in all three groups was 65.1–66.0 *(p* < 0.001), which is higher than 61, the mean age of the non-pain group (*p* < 0.001). According to the pain site, the mean age for the hip joint pain group was 66.0±0.46, the knee joint pain group was 65.1±0.36, the lower back pain group was 65.7±0.32, and the order of mean ages was hip joint pain group > lower back pain group > knee joint pain group (*p* < 0.001). When comparing the sexes, more than 72% of the hip joint, knee joint, and lower back pain groups were female. In terms of educational level, for the hip joint pain group, regardless of the pain status, the percentage was highest for “college graduate or higher” at 60.8% for the pain group and 36.5% for the non-pain group. Referring to the knee joint and lower back group, regardless of the pain status, the percentage of “elementary school graduate or lower” was the highest for the knee joint pain group at 59.6%, the non-knee joint pain group at 33.5%, the lower back pain group at 57.7%, and the non-lower back pain group at 33.6%. These findings indicate that there was a significant difference in educational level in relation to the pain sites (p < 0.001). Income level was classified in the order of “low”>“low–moderate”>“moderate–high”>“high” for the pain group; for the non-pain group, the ratio of the quartile income level was similar, in the range of 23–27% (p < 0.001).

Second, considering health-related characteristics, for BMI values, a “normal” BMI accounted for the highest numbers in both the pain group and the non-pain group (hip joint p = 0.129, knee joint p < 0.001, and lower back p = 0.031). When comparing the smoking status of the subjects, the percentage of non-smokers was 50% or more in both the pain and non-pain groups (p < 0.001). In the case of alcohol consumption, the proportion of the answer “none” was the highest in both the pain and non-pain groups (p < 0.001). Referring to comorbid diseases, regardless of pain status, the percentage of subjects without angina, diabetes mellitus, hypertension, and rheumatoid arthritis was significantly higher in all groups; specifically, the proportion of subjects without rheumatoid arthritis was 92% or higher in all groups (p < 0.001).

#### Comparison of mental health and quality of life between pain and non-pain groups by sex

The comparison of mental health and quality of life between the pain group and the non-pain group was analyzed according to sex (Table 2). When comparing the differences according to sex, the response rate of “Yes” to stress was higher for females in the pain group than for males. There was no significant difference according to the pain sites (*p* < 0.05). Referring to “Depression,” the “yes” response rate was higher for females in the pain group than for males. There was no significant difference between the pain sites (*p* < 0.05). Among the quality of life items, referring to “Mobility,” males with OA were shown to have a problem with Mobility in the order of knee joint (53.0%) > lower back (51.6%) > hip joint (43.7%; *p* < 0.001), while females with OA were shown to have Mobility issues in the order of lower back (49.0%) > knee joint (44.6%) > hip joint (41.8%; *p* < 0.001). In the “Self-care” category, males with OA were found to have problems with Self-care in the order of knee joint (84.3%) > lower back (83.0%) > hip joint (72.3%), while for females, the order was lower back (85.0%) > knee joint (84.3%) > hip joint (81.2%)(*p* < 0.001). In the “Usual activities” category, males in the knee joint pain group and lower back pain group had a higher than 50% rate of “No” responses, regardless of pain status (*p* < 0.001). In contrast, in the group with hip joint pain, the “Yes” response rate was 51.2%, compared with the “No” response rate (*p* < 0.001). The results show that both males and females with OA had problems with “Usual activities” in the order of knee joint (male: 67.8%, female: 63.7%) > lower back (male: 66.7%, female: 63.5%) > hip joint (male: 48.8%, female: 56.5%; *p* < 0.001). In the “Pain/discomfort” category, males experienced pain and discomfort in the order of hip joint (71.6%) > lower back (60.2%) > knee joint (57.1%); for females, the order was hip joint (70.4%) > knee joint (66.8%) > lower back (64.0%; *p* < 0.001). Considering the “Anxiety/depression” category, both males and females with OA responded that they experienced anxiety or depression in the order of hip joint (male: 30.2%, female: 35.6%) > lower back (male: 24.5%, female: 31.0%) > knee joint (male: 23.2%, female: 30.2%; *p* < 0.001).

**Table 2:** Characteristics of the mental health and quality of life according to the osteoarthritis pain site and sex.

Category	Hip joint pain	Non-hip joint pain	P-value	Knee joint pain	Non-Knee joint pain	P-value	Low back pain	Non-Low back pain		P-value
Male	(n = 122)	(n = 2186)		(n = 289)	(n = 2019)		(n = 328)	(n = 1980)		
**Stress**										
No	89(74.2)	1844(83.2)	0.029*	217(76.1)	1716(83.7)	0.005**	240(71.2)	1693(84.5)		p<0.001
Yes	33(25.8)	342(16.8)		72(23.9)	303(16.3)		88(28.8)	287(15.5)		
**Depression**										
No	101(85.0)	1968(90.7)	0.064	236(81.8)	1833(91.6)	p<0.001	265(80.4)	1804(91.9)		p<0.001
Yes	21(15.0)	218(9.3)		53(18.2)	186(8.4)		63(19.6)	176(8.1)		
**Mobility**										
No	52(43.7)	1803(83.9)	p<0.001	147(53.0)	1708(86.1)	p<0.001	171(51.6)	1684(86.6)		p<0.001
Yes	70(56.3)	383(16.1)		142(47.0)	311(13.9)		157(48.4)	296(13.4)		
**Self-care**										
No	86(72.3)	2062(94.6)	p<0.001	241(84.3)	1907(94.8)	p<0.001	272(83.0)	1876(95.1)		p<0.001
Yes	36(27.7)	124(5.4)		48(15.7)	112(5.2)		56(17.0)	104(4.9)		
**Usual activities**										
No	60(48.8)	1984(91.0)	p<0.001	195(67.8)	1849(92.1)	p<0.001	224(66.7)	1820(92.4)		p<0.001
Yes	62(51.2)	202(9.0)		94(32.2)	170(7.9)		104(33.3)	160(7.6)		
**Pain/discomfort**										
No	34(28.1)	1702(78.6)	p<0.001	127(42.9)	1609(80.9)	p<0.001	130(39.8)	1606(81.7)		p<0.001
Yes	88(71.9)	484(21.4)		162(57.1)	410(19.1)		198(60.2)	364(18.3)		
**Anxiety/depression**										
No	82(69.8)	1978(90.4)	p<0.001	225(76.8)	1835(91.2)	p<0.001	244(75.5)	1816(91.6)		p<0.001
Yes	40(30.2)	208(9.6)		64(23.2)	184(8.8)		84(24.5)	164(8.4)		
**Female**	**(n = 434)**	**(n = 2659)**		**(n = 868)**	**(n = 2225)**		**(n = 918)**	**(n = 2175)**		
**Stress**										
No	282(65)	2063(76.6)	p<0.001	570(65.1)	1775(78.9)	p<0.001	619(66.5)	1726(78.5)		p<0.001
Yes	152(35)	596(23.4)		298(34.9)	450(21.1)		299(33.5)	449(21.5)		
**Depression**										
No	315(73)	2211(83.2)	p<0.001	638(73.2)	1888(85.1)	p<0.001	684(73.9)	1842(85.1)		p<0.001
Yes	119(27)	448(16.8)		230(26.8)	337(14.9)		234(26.1)	333(14.9)		
**Mobility**										
No	172(41.8)	1985(76.5)	p<0.001	369(44.6)	1788(82.1)	p<0.001	416(49.0)	1741(81.1)		p<0.001
Yes	262(58.2)	674(23.5)		499(55.4)	437(17.9)		502(51.0)	434(18.9)		
**Self-care**										
No	346(81.2)	2475(93.8)	p<0.001	717(84.3)	2104(95.1)	p<0.001	766(85.0)	2055(95.0)		p<0.001
Yes	88(18.8)	184(6.2)		151(15.7)	121(4.9)		152(15.0)	120(5.0)		
**Usual activities**										
No	240(56.5)	2260(85.8)	p<0.001	540(63.7)	1960(88.8)	p<0.001	566(63.5)	1934(89.3)		p<0.001
Yes	194(43.5)	399(14.2)		328(36.3)	265(11.2)		352(36.5)	241(10.7)		
**Pain/discomfort**										
No	122(29.6)	1780(67.6)	p<0.001	278(33.2)	1624(73.5)	p<0.001	319(36.0)	1583(73.2)		p<0.001
Yes	312(70.4)	879(32.4)		590(66.8)	601(26.5)		599(64.0)	592(26.8)		
**Anxiety/depression**										
No	276(64.4)	2234(84.4)	p<0.001	601(69.8)	1909(86.2)	p<0.001	628(69.0)	1882(86.8)		p<0.001
Yes	158(35.6)	425(15.6)		267(30.2)	316(13.8)		290(31.0)	293(13.2)		

Values are presented as number (%). P-values were calculated using Chi-square test

*p<0.05

**p<0.001.

#### Association between OA pain sites and mental health

Table 3 presents how mental health is affected by the OA pain site. Model 1 is an unadjusted model and Model 2 has been adjusted for age, BMI, and other environmental factors. Mental health items comprise stress and depression. Overall, the pain group experienced more stress and depression than the non-pain group (*p* < 0.05). Based on Model 2, for males, the stress category showed results in the order of lower back pain group (OR 2.30, 95% CI 1.68–3.15) > hip joint pain group (OR 1.83, 95% CI 1.13–2.96) > knee joint pain group (OR 1.65, 95% CI 1.17–2.33). This indicates that they experience more stress than the non-pain group (*p* < 0.05). In terms of OA pain sites, the subjects were vulnerable to stress in the order of lower back (OR 2.30, 95% CI 1.68–3.15) > hip joint (OR 1.83, 95% CI 1.13–2.96) > knee joint (OR 1.65, 95% CI 1.17–2.33). In the depression category, pain groups experienced more depression than the non-pain groups in the order of lower back pain group (OR 2.42, 95% CI 1.66–3.52) > knee joint pain group (OR 2.10, 95% CI 1.40–3.13) > hip joint pain group (OR 1.50, 95% CI 0.87–2.57; *p* < 0.05). Considering the OA pain sites, the subjects were vulnerable to stress in the order of lower back (OR 2.42, 95% CI 1.66–3.52) > knee joint (OR 2.10, 95% CI 1.40–3.13) > hip joint (OR 1.50, 95% CI 0.87–2.57; *p* < 0.05). In conclusion, male people with OA experience the most stress and depression when the pain site is lower back (*p* < 0.05).

**Table 3:** Association between mental health and OA pain site using multiple logistic regression analysis.

		Stress				Depression			
		Model 1		Model 2		Model 1		Model 2	
		OR(95% CI)	*P*-value	OR(95% CI)	*P*-value	OR(95% CI)	*P*-value	OR(95% CI)	*P*-value
**Male**	**Hip joint pain**	1.71 (1.71–2.72)	0.023*	1.83 (1.13–2.96)	0.013*	1.71 (0.99–2.92)	0.051	1.50 (0.87–2.57)	0.141
	**Knee joint pain**	1.61 (1.17–2.22)	0.004*	1.65 (1.17–2.33)	0.004**	2.41 (1.64–3.55)	p<0.001	2.10 (1.40–3.13)	p<0.001
	**Low back pain**	2.20 (1.60–3.03)	p<0.001	2.30 (1.68–3.15)	p<0.001	2.75 (1.92–3.95)	p<0.001	2.42 (1.66–3.52)	p<0.001
**Female**	**Hip joint pain**	1.76 (1.38–2.25)	p<0.001	1.64 (1.27–2.13)	p<0.001	1.83 (1.36–2.46)	p<0.001	1.61 (1.19–2.17)	0.002**
	**Knee joint pain**	2.00 (1.64–2.44)	p<0.001	1.99 (1.61–2.44)	p<0.001	2.08 (1.67–2.60)	p<0.001	1.91 (1.52–2.41)	p<0.001
	**Low back pain**	1.84 (1.51–2.24)	p<0.001	1.77 (1.46–2.15)	p<0.001	2.02 (1.62–2.51)	p<0.001	1.84 (1.48–2.30)	p<0.001

OR, indicates odds ratio; 95% CI, 95% confidence interval. Model 1 was unadjusted odds ratio. Model 2 was fully adjusted by age, BMI and other environmental factors such as smoking, alcohol consumption, household income, educational level, and comorbidities.

*p<0.05

**p<0.001.

Based on Model 2, in the case of females, the pain group results for the stress category were in the order of knee joint pain group (OR 1.99, 95% CI 1.61–2.44) > lower back pain group (OR 1.77, 95% CI 1.46–2.15) > hip joint pain group (OR 1.61, 95% CI 1.27–2.13). This indicates that the pain groups experienced more stress, compared with non-pain groups (*p* < 0.05). In the depression category, the order of knee joint pain group (OR 1.91, 95% CI 1.52–2.41) > lower back pain group (OR 1.84, 95% CI 1.487–2.30) > hip joint pain group (OR 1.61, 95% CI 1.19–2.17) was the same. These results indicate that the pain groups experienced more depression than the non-pain groups (*p* < 0.05). Summarizing the findings for female subjects, female people with OA showed the most stress and depression in case of knee joint pain site, followed by lower back and hip (*p* < 0.05).

#### Association between OA pain sites and quality of life in male

Table 4 outlines the association between OA pain sites and quality of life in males. Model 1 is an unadjusted model and Model 2 has been adjusted for age, BMI, and other environmental factors. There are five quality of life items: “Mobility,” “Self-care,” “Usual activities,” “Pain/discomfort,” and “Anxiety/depression.” The pain group had higher percentages for all five items than the non-pain group. For Model 1, in all five categories, the hip joint pain group showed the highest values for Mobility (OR 6.70, 95% CI 4.33–10.3), Self-care (OR 6.67, 95% CI 4.19–10.61), Usual activities (OR 10.63, 95% CI 6.85–16.48), Pain/discomfort (OR 9.40, 95% CI 5.90–14.99), and Anxiety/depression (OR 4.07, 95% CI 2.57–6.44) in that order, compared to the non-pain group, indicating higher values than those of knee joint or lower back pain groups compared to their respective non-pain groups (*p* < 0.001). For Model 2, the hip joint pain group showed the highest odds ratio in their responses regarding the quality of life, compared with those of the knee joint or lower back pain groups (*p* < 0.001). In Model 2, the hip joint pain group showed the highest odds ratio for Usual activities (OR 11.51, 95% CI 6.77–19.55), followed by Pain/discomfort (OR 8.18, 95% CI 5.05–13.25; *p* < 0.001). The knee joint pain group showed the highest odds ratio for Mobility (OR 4.81, 95% CI 3.38–6.83) and lower back pain group showed the highest odds ratio for Pain/discomfort (OR 5.86, 95% CI 4.22–8.13; *p* < 0.001). Considering each EQ-5D category, the order of highest to lowest values was as follows: Mobility at hip joint > lower back > knee joint, Self-care at hip joint > knee joint > lower back, Usual activities at hip joint > lower back > knee joint, Pain/discomfort at hip joint > lower back > knee joint, and Anxiety/depression at hip joint > lower back > knee joint (*p* < 0.001). Except for Self-care, our analysis shows that all other categories experienced problems in the order of hip joint > lower back > knee joint (*p* < 0.001).

**Table 4:** Association between quality of life and OA pain site using multiple logistic regression analysis by male.

	Model 1		Model 2	
	OR(95% CI)	*P*-value	OR(95% CI)	*P*-value
**Mobility**				
Hip joint pain	6.70(4.33–10.38)	p<0.001	6.12(3.54–10.58)	p<0.001
Knee joint pain	5.47(4.06–7.37)	p<0.001	4.81(3.38–6.83)	p<0.001
Low back pain	6.05(4.57–8.01)	p<0.001	5.04(3.72–6.83)	p<0.001
**Self-care**				
Hip joint pain	6.67(4.19–10.61)	p<0.001	6.11(3.68–10.13)	p<0.001
Knee joint pain	3.41(2.17–5.35)	p<0.001	2.64(1.61–4.33)	p<0.001
Low back pain	3.99(2.69–5.92)	p<0.001	2.62(1.66–4.12)	p<0.001
**Usual activities**				
Hip joint pain	10.63(6.85–16.48)	p<0.001	11.51(6.77–19.55)	p<0.001
Knee joint pain	5.50(4.00–7.58)	p<0.001	4.64(3.19–6.75)	p<0.001
Low back pain	6.10(4.50–8.26)	p<0.001	4.72(3.32–6.71)	p<0.001
**Pain/discomfort**				
Hip joint pain	9.40(5.90–14.99)	p<0.001	8.18(5.05–13.25)	p<0.001
Knee joint pain	5.65(4.18–7.65)	p<0.001	4.67(3.38–6.44)	p<0.001
Low back pain	6.75(4.95–9.20)	p<0.001	5.86(4.22–8.13)	p<0.001
**Anxiety/depression**				
Hip joint pain	4.07(2.57–6.44)	p<0.001	3.93(2.44–6.32)	p<0.001
Knee joint pain	3.15(2.16–4.58)	p<0.001	2.62(1.75–3.93)	p<0.001
Low back pain	3.52(2.49–4.98)	p<0.001	2.74(1.92–3.89)	p<0.001

OR, indicates odds ratio; 95% CI, 95% confidence interval. Model 1 was unadjusted OR. Model 2 was fully adjusted by age, BMI and other environmental factors such as smoking, alcohol consumption, household income, educational level, and comorbidities.

*p<0.05

**p<0.001.

#### Association between OA pain sites and quality of life in female

Table 5 outlines the association between OA pain sites and quality of life in females. Model 1 is an unadjusted model and Model 2 has been adjusted for age, BMI, and other environmental factors.” In both Model 1 and Model 2, the pain group showed a higher odds ratio than the non-pain group in all five categories (*p* < 0.001). Regarding Model 2, for all pain groups of knee joint, hip joint, and lower back, Pain/discomfort was the highest among the five EQ-5D categories in the order of knee joint (OR 4.87, 95% CI 3.98–5.97) > hip joint (OR 4.21 95% CI 3.22–5.51) > lower back (OR 4.21, 95% CI 3.49–5.09; *p* < 0.001). In terms of the quality of life categories, the order of difficulties in each item was knee joint > hip joint > lower back for Mobility, knee joint > hip joint > lower back for Self-care, lower back > hip joint > knee joint for Usual activities, knee joint > hip joint = lower back for Pain/discomfort, and lower back > hip joint > knee joint for anxiety/depression (*p* < 0.001).

**Table 5:** Association between quality of life and OA pain site using multiple logistic regression analysis by female.

	Model 1		Model 2	
	OR(95% CI)	*P*-value	OR(95% CI)	*P*-value
**Mobility**				
Hip joint pain	4.53(3.95–5.72)	p<0.001	3.56(2.68–4.71)	p<0.001
Knee joint pain	5.70(4.69–6.93)	p<0.001	4.40(3.53–5.48)	p<0.001
Low back pain	4.46(3.72–5.34)	p<0.001	3.28(2.70–3.98)	p<0.001
**Self-care**				
Hip joint pain	3.48(2.59–4.96)	p<0.001	2.29(1.63–3.22)	p<0.001
Knee joint pain	3.58(2.71–4.73)	p<0.001	2.50(1.85–3.38)	p<0.001
Low back pain	3.33(2.49–4.46)	p<0.001	2.07(1.52–2.81)	p<0.001
**Usual activities**				
Hip joint pain	4.66(3.64–5.98)	p<0.001	3.57(2.68–4.77)	p<0.001
Knee joint pain	4.52(3.60–5.69)	p<0.001	3.39(2.64–4.35)	p<0.001
Low back pain	4.82(3.95–5.89)	p<0.001	3.62(2.93–4.48)	p<0.001
**Pain/discomfort**				
Hip joint pain	4.97(3.84–6.43)	p<0.001	4.21(3.22–5.51)	p<0.001
Knee joint pain	5.57(4.95–6.77)	p<0.001	4.87(3.98–5.97)	p<0.001
Low back pain	4.84(4.02–5.83)	p<0.001	4.21(3.49–5.09)	p<0.001
**Anxiety/depression**				
Hip joint pain	2.98(2.32–3.82)	p<0.001	2.49(1.91–3.24)	p<0.001
Knee joint pain	2.70(2.20–3.30)	p<0.001	2.38(1.92–2.96)	p<0.001
Low back pain	2.95(2.36–3.70)	p<0.001	2.64(2.08–3.34)	p<0.001

OR, indicates odds ratio; 95% CI, 95% confidence interval. Model 1 was unadjusted OR. Model 2 was fully adjusted by age, BMI and other environmental factors such as smoking, alcohol consumption, household income, educational level, and comorbidities.

*p<0.05

**p<0.001.

## Discussion

Osteoarthritis (OA) is a representative degenerative chronic disease that commonly occurs in synovial joints, with clinical manifestations of joint pain, stiffness, decreased range of motion, and crepitus. OA occurs mainly in sites such as hip and knee joints and the lower back. These sites are subject to high pressure from weight loading, which causes degenerative changes in the lower cartilage [19]. Approximately 10.7% of the Korean population suffers from OA, and its prevalence shows a sharp increase with age [20]. OA accounts for the largest proportion of medical expenditure incurred from chronic disease [21] and it is predicted that the prevalence of OA—and therefore its related medical expenditure—will increase with the progression of today’s aging society. It can therefore be said that OA will cause considerable financial burden. Although OA is not regarded as life-threatening, people with OA experience persistent pain and stiffness in their daily lives, leading not only to physical disabilities but also to mental health issues such as secondary depression and anxiety as well as decreased quality of life [4,6]. The health-related quality of life of older adults with OA has been shown to be lower than that of older adults without OA, and quality of life tends to deteriorate with age. In addition, older adults with OA perceive their general health to be worse than those without OA and experience more depression. OA has been reported to have a higher prevalence of associated depression than other diseases [22]. In South Korea, the prevalence of depression in people with OA is 11.2%, higher than that of other conditions such as hypertension (8.3%), diabetes mellitus (8.7%), and cancer (11.1%); furthermore, the likelihood of depression is 1.5 times higher for people with OA than for those without OA [23]. This pattern of persistent depression can increase the risk of suicide; in fact, instances of suicide in people with OA are 1.5 times higher than in the general population [24]. The main reason why people with OA experience depression is that they face physical difficulties and social isolation due to chronic pain and gait disturbance, and their stress levels rise due to economic and psychological burden because of the recurrence and exacerbation of symptoms [3,7]. Although OA is difficult to cure, it is possible to improve health-related quality of life by controlling the symptoms of the disease through continuous management and Self-care. In prior studies on people with OA, smokers were found to have lower health-related quality of life than non-smokers, and drinkers compared with non-drinkers [25,26]. As can be seen from the findings of this study as well as prior studies, chronic diseases, obesity, exercise, alcohol consumption, and smoking have been identified as risk factors affecting OA. Therefore, it can be said that higher treatment satisfaction is possible when treatment is performed while considering not only the OA symptoms themselves, but also other environmental factors.

Currently, most OA-related studies focus primarily on the prevalence and risk factors of OA, and there have been few studies related to the quality of life and mental health of people with OA. Factors such as depression affect OA symptoms themselves [27], aggravating pain and increasing the risk of suicide. It is clear that there are sufficient valid reasons to examine the health-related quality of life of people suffering from a disease such as OA, a disease with high prevalence that requires continuous medical services and have high medical expenses. Domestic studies on the association between quality of life and OA include topics such as the impact of difficulties in the activities of daily living, pain and the actual practice of exercise on the quality of life for people with OA in rural areas [28], and investigations of the relationship between health status, health behavior, and health-related quality of life in older adults with OA [29]. However, there have not been many studies on the association between mental health and quality of life according to the OA sites.

This study was conducted to analyze the association between quality of life and mental health according to OA sites using KNHANES-VI data. The OA sites used in this study were hip joint, knee joint, and lower back, which are regions with high OA frequency.

Our comparison of mental health and quality of life between the pain and non-pain groups by sex (Table 2) showed that females with OA had higher rates of experiencing stress and depression than males. This is consistent with the results of previous studies showing differences in the prevalence of depression in patients with OA by sex [27]. Females with OA (12.2%) reported a higher incidence of depression than males (7.6%), and previous studies involving older adults with OA also showed that depression incidence in female older adults (35.3%) was higher than in males (29.7%) [16,24]. The reason for females experiencing more depression than males may be due to various factors—such as hormones, postpartum depression, role stress, socialization, and social status—being involved, in addition to the depression related to the disease itself [30]. OA itself can cause depression, but depression can also cause and exacerbate OA. Results of a previous study showed that the risk of OA was reduced by 0.62 times in elderly females without depression [27]. Therefore, in the future, when treating female patients with OA, a mental health treatment plan that reduces depression is essential as part of the treatment plan for OA.

According to the model analysis of the association between OA pain sites and mental health (Table 3), first, we can see that the pain group experiences more stress and depression than the non-pain group. For males with OA, stress is in the order of lower back (OR 2.30, 95% CI 1.68–3.15) > hip joint (OR 1.83, 95% CI 1.13–2.96) > knee (OR 1.65, 95% CI 1.17–2.33). Furthermore, they were vulnerable to depression in the order of lower back (OR 2.42, 95% CI 1.66–3.52) > knee (OR 2.10, 95% CI 1.40–3.13) > hip joint (OR 1.50, 95% CI 0.87–2.57; *p* < 0.05). Considering females with OA, the rate of stress and depression was higher in the order of knee(Stress OR 1.99, 95% CI 1.61–2.44, Depression OR 1.91, 95% CI 1.52–2.41) > lower back(Stress OR 1.77, 95% CI 1.46–2.15, Depression OR 1.84, 95% CI 1.48–2.30) > hip(Stress OR 1.64, CI 1.27–2.13, Depression OR 1.61, 95% CI 1.19–2.17))(*p* < 0.05). This is consistent with the results of previous studies in which knee pain was reported as an influencing factor of depression in female patients with OA, the status of knee stiffness and decreased range of motion negatively affected health-related quality of life, and depression in females with knee pain was significantly higher [7,11,31]. The results of this study therefore show that in people with OA, the levels of stress and depression varies according to sex and pain sites; specifically, a higher proportion of female patients was shown to experience stress and depression, compared with male patients (*p* < 0.05).

The EQ-5D, which was used to assess the quality of life in KNHANES, was also used in this study, as it offers convenience and high validity in terms of the assessment of health-related quality of life in people with OA [18]. In the EQ-5D, a total of five categories were assessed: Mobility, Self-care, Usual activities, Pain/discomfort, and Anxiety/depression. According to the model analysis between the pain sites and quality of life by sex (Table 4), the hip joint pain group showed the highest odds ratio in all categories of EQ-5D for male patients with OA (*p* < 0.001). In particular, the highest odds ratio found was for Usual activities (OR 11.51, 95% CI 6.77–19.55), and all categories except Self-care showed high odds ratios for the order of hip joint > lower back > knee joint (*p* < 0.001). This means that for males, it was found that, among OA pain sites, the hip joint is the most significant factor in determining quality of life. This is consistent with the findings of a previous study, which reported that if hip joint pain becomes chronic, it causes limitations in everyday life, such as sitting and standing up. This, in turn, limits activities of daily living and adversely affects individuals and households by degrading individuals’ quality of life [32,33]. Looking at Table 5, in females with OA, the knee pain group experienced more difficulties in Mobility, Self-care, and Pain/discomfort than the hip joint and lower back pain groups. This is also consistent with the above-mentioned finding that knee joint pain affects depression in female patients with OA. The prevalence of knee OA also increases with age; specifically, 11% of females aged 60 or older have OA of the knee [34]. In addition, according to Jinks et al., for patients aged 50 or older who complain of knee joint pain, depression was reported as one of the factors associated with severe functional disabilities along with status of chronic condition, age (> 75 years), bilateral injury, and BMI (>30) [35]. It can therefore be said that for female patients with knee OA, the quality of life should always be assessed and included in treatment plans and objectives. Moreover, in females’ EQ-5D categories, Pain/discomfort items showed higher OR values for the knee joint, hip joint, and lower back pain groups, compared with other categories. An existing study of 9,476 older female patients diagnosed with OA using raw data from the 2009 Korean Community Health Survey reported that more than half of them reported stress levels of “severe or higher” and that the group experiencing severe stress was more depressed [36], which is consistent with the findings of this study. When considering the results comprehensively, hip joint pain greatly affects the mental health and quality of life of male patients with OA, while for female patients, knee joint pain has a great impact on mental health and quality of life. A prior study of patients with hip joint and knee joint pain showed a significant effect with loss of range of motion (46.2%) and the level of effect on emotion (30%) [37] while in other studies, patients with hip and knee OA showed lower values of physical and psychological health-related quality of life than the non-pain group, which is consistent with the findings of this study [38,39].

It has been reported that the health-related quality of life of older adults with OA is lower in both physical and mental aspects than those without OA; in particular, that the quality of life related to mental health is approximately twice as low as that of healthy older adults [40]. This is because chronic pain, gait disturbance, and disabilities in daily lives due to OA causes depression. Considering mental health and depression, deteriorating health-related quality of life can lead to suicide among older adults [13]; therefore, special attention and care are needed. Patients with chronic musculoskeletal pain have been reported to experience more severe pain and disabilities when emotional factors such as depression or anxiety are combined with the disease [41]. In this study, the pain groups had a higher levels of stress and depression than the non-pain groups and their quality of life was lower. In particular, male patients showed higher values in the hip joint pain group and female patients in the knee joint pain group. In view of previous studies as well as the findings of this study, it is necessary to focus on emotional symptoms and signs, such as stress and depression, while also considering the differences in sex and OA pain sites during OA treatment, and monitor those symptoms and signs on a regular basis.

This study used cross-sectional survey data of KNHANES-VI, which are highly reliable data on a national scale, and is the first study to explain the association between mental health and quality of life according to OA pain sites. The difference in mental health and quality of life according to OA pain site is not large; however, the significance of the study lies in that it examines mental health and quality of life according to the different pain sites. In addition, as factors affecting the mental health and quality of life according to OA pain sites and sex were identified, individual treatment plans can be designed according to sex and pain sites as treatment for OA. This study had certain limitations. Although this study identified the association of mental health and quality of life according to the OA pain sites, the correlation between them has not been identified. This study has not considered the possibilities and cases of OA occurrence in multiple sites; furthermore, the number of OA pain sites has not been considered. In fact, in a prior study on older adults with OA in rural areas, it was reported that the number of pain sites and limitation in activities of daily living were the influencing factors of the health-related quality of life, rather than the pain itself [28]. Therefore, studies related to the combination of OA pains or the number of pain sites are required in the future. In addition, it is also necessary to investigate the correlation between mental health and quality of life according to the pain sites for each age group. In the case of depression, this study investigated only the presence of depression symptoms and did not assess the level of depression. The level of depression also needs to be accurately assessed; therefore, it is necessary to use a depression assessment scale such as CES D-10 (Centre for Epidemiological Studies Depression Scale) in the future. In studies in other countries, survey tools to assess the quality of life of patients with OA have been developed and utilized [42]. Currently, there are three types of Korean survey tools available: Health Assessment Questionnaire (HAQ), the Western Ontario and Mac Master Universities OA Index (WOMAC), and the Arthritis Impact Measurement Scale 2 (AIMS2) [42]. Although these survey tools were not utilized in this study, OA-related questionnaires should be utilized more in future research and treatment. As this is a cross-sectional study, the associations between OA pain, mental health, and quality of life were evaluated using OR values, without analyzing the causal relationship between these variables. Moreover, as it was applied to the population aged 50 years and older, those under 50 were excluded from the analysis. As the self-report type questionnaire for respective assessment of scores for OA pain, mental health, and quality of life, the possibility of response bias cannot be ruled out.

## Conclusion

The results of this study show that there was a strong association between mental health and quality of life and the pain sites of people suffering from OA. When comparing the sexes, a higher proportion of females with OA experienced stress and depression than males. In addition, in terms of quality of life, hip joint pain in males and knee joint pain in females were found to be the site that most significantly affected quality of life. Therefore, when determining OA pain, we present the pain site, sex, mental health status, and quality of life as independent risk factors.
